# Memory T cell proliferative responses and IFN-γ productivity sustain long-lasting efficacy of a Cap-based PCV2 vaccine upon PCV2 natural infection and associated disease

**DOI:** 10.1186/1297-9716-45-44

**Published:** 2014-04-16

**Authors:** Luca Ferrari, Paolo Borghetti, Elena De Angelis, Paolo Martelli

**Affiliations:** 1 Department of Veterinary Science, University of Parma, Via del Taglio, 10, 43126 Parma, Italy

## Abstract

Porcine circovirus type 2 (PCV2) vaccination represents an important measure to cope with PCV2 infection; however, data regarding the modulation of the immune cell compartment are still limited, especially under field conditions. This study is aimed at investigating the features of the cellular immune response in conventional piglets induced by vaccination using a capsid (Cap) protein-based PCV2 vaccine compared to unvaccinated animals when exposed to PCV2 natural infection. Immune reactivity was evaluated by quantifying peripheral cell subsets involved in the anti-viral response and characterizing the interferon-gamma (IFN-γ) secreting cell (SC) responsiveness both in vivo and upon in vitro whole PCV2 recall. The vaccination triggered an early and intense IFN-γ secreting cell response and induced the activation of peripheral lymphocytes. The early increase of IFN-γ SC frequencies resulted in a remarkable and transient tendency to increased IFN-γ productivity in vaccinated pigs. In vaccinated animals, soon before the onset of infection occurred 15-16 weeks post-vaccination, the recalled PCV2-specific immune response was characterized by moderate PCV2-specific IFN-γ secreting cell frequencies and augmented productivity together with reactive CD4^+^CD8^+^ memory T cells. Conversely, upon infection, unvaccinated animals showed very high frequencies of IFN-γ secreting cells and a tendency to lower productivity, which paralleled with effector CD4^–^CD8^+^ cytotoxic cell responsiveness. The study shows that PCV2 vaccination induces a long-lasting immunity sustained by memory T cells and IFN-γ secreting cells that potentially played a role in preventing the onset of infection; the extent and duration of this reactivity can be an important feature for evaluating the protective immunity induced by vaccination.

## Introduction

Porcine circovirus type 2 (PCV2) is one of the main pathogens responsible for relevant economic losses worldwide; this small single-strand DNA virus belongs to the *Circoviridae* family and is the causative infectious agent, together with other viral and bacterial pathogens, of the so-called porcine circovirus associated diseases (PCVD) [1,2]. Several in vitro and in vivo studies have confirmed that the virus interacts and modulates several components of the pig immune system, having its major tropism to the cells of the monocyte/macrophage lineage (MØ) and dendritic cells (DC). However, it seems that no efficient replication and spread derives from infection of these susceptible immune cells, thus sustaining persistent infection. In fact, PCV2 replication is dependent on the activation and proliferation of susceptible cells since the virus lacks its own polymerase for autonomous propagation. It was also demonstrated that lymphocytes, especially if activated to lymphoblasts, can carry PCV2 antigens and genome, thus representing additional target cells. The interaction with innate immune cells and/or with lymphocytes/lymphoblasts triggers virus replication and subsequently immune responses in tissues; PCV2 can also be recovered from peripheral blood mononuclear cells (PBMC), specifically from T and B lymphocytes [3-5].

Recent studies demonstrated that the onset of humoral immunity as total and virus-neutralizing antibodies (VNA) upon PCV2 natural or experimental infection is an important response to counteract the onset of clinical signs. In parallel, cellular responses such as DC- and T cell-derived cytokine production (e.g. IL-1β, IL-8, TNF-α, IL-12, IFN-γ, IL-10) are modulated during early and late phases of infection both peripherally and locally in primary/secondary lymphoid organs [6-17]. Diseased pigs can show the increase of peripheral SWC3^+^ monocytes [18] or neutrophils [19] and reduction of the leukocyte/lymphocyte population specifically involving CD3^+^, CD4^+^, CD8^+^, memory T helper (Th) CD4^+^CD8^+^ cells and CD21^+^/IgM^+^ B cells [12,18,20], besides increased levels of IL-10 and virus-specific IL-10 secreting cells in PBMC and lymphoid organs [7,8,10,21]. Nonetheless, cellular changes are not univocal due to the variable onset and development of the disease possibly related to the status and age of the infected animals.

On the contrary, since PCVD is hardly induced experimentally due to the absence of triggering pathogenic co-factors, several studies have been performed under controlled conditions in which PCV2 infection sustained only subclinical outcomes. In some cases, PCV2 infection elicited an antibody response together with virus-specific IFN-γ secreting cells constituted by CD4^+^ and CD8^+^ cells [13,17,22-24]. In non-diseased pigs, the changes of immune cell subsets such as T cells (naïve/memory T helper, γ/δ T and cytotoxic T lymphocytes (CTL)) and B cells are not intense and can be sporadic [17,25]. However, scarce data are available during PCV2 natural infection, especially in relation to vaccination, which surely represents one of the major measures to control PCVD. Current commercial vaccines proved to be efficacious in reducing mortality and morbidity, and pathological lesions, but the immune activation induced systemically and at tissue level has not been thoroughly investigated. The immune response evaluated under experimental and field conditions highlighted the involvement of both humoral and cellular immune responses in sustaining clinical protection. Specifically, studies on the efficacy of experimental/commercial vaccines in SPF (specific pathogen-free) or conventional animals experimentally challenged after 3-6 weeks with PCV2 strains showed the induction of virus-specific antibodies and IFN-γ secreting cells associated with the reduction of viremia, shedding and viral burden in tissues upon subclinical outcomes [23,26-33]. A study by Seo et al. showed that vaccination of conventional-experimentally infected piglets using an inactivated chimeric PCV1-2 vaccine induced increases of CD3^+^ and CD4^+^ cells in the blood and sustained higher CD4^+^ cell levels after infection [34].

The present study is aimed at providing further insight into the features of the cellular immune response elicited in PCV2-vaccinated and non-vaccinated pigs naturally infected by PCV2 at 15-16 weeks post-vaccination. The reactivity of the immune cells was evaluated and characterized in terms of changes of peripheral T immune subsets, in vitro modulation of T cells upon stimulation with PCV2 or PCV2 + mitogen and in-depth combined quantitative and qualitative analysis of the virus-specific IFN-γ secreting cell response.

## Materials and methods

### Animals and experimental design

The animals enrolled in this field study were selected in a farrow-to-finish herd with a history of clinical signs and mortality related to PCVD in pigs older than 15 weeks due to a PCV2b strain.

The details about the herd health status and clinical outcome are reported in Martelli et al. [29]. At inclusion, the farm was seronegative for Aujeszky’s disease virus (ADV) and seropositive for PCV2, porcine reproductive and respiratory syndrome virus (PRRSV), *Mycoplasma hyopneumoniae* (*M. hyopneumoniae*) and *Actinobacillus pleuropneumoniae* (*A. pleuropneumoniae*). PCV2 seropositivity in sows was due to a previous infection and not to vaccination. Some animals had antibodies against swine influenza virus (SIV). Piglets were vaccinated for ADV according to the National Control Program and for *M. hyopneumoniae* at 1 week of age (one shot vaccination).

At weaning (3 weeks of age), two groups of piglets were designated as PCV2-vaccinated (PCV2-vac) group (200 piglets) and control (C) unvaccinated group (200 piglets), respectively. Vaccination was performed intramuscularly (IM) by inoculating a single dose (2 mL) of a PCV2 vaccine suspended in adjuvant to PCV2-vac pigs while the same volume of adjuvant only was administered to controls.

Thirty animals were selected for the study from the PCV2-vac group (10 pigs) and C group (20 pigs). Blood samples were collected at 3, 4, 5, 6, 7 and 9 weeks of age (respectively 0, 1, 2, 3, 4 and 6 weeks post-vaccination (PV)). Afterwards, the pigs were naturally exposed to PCV2 infection by commingling them with unvaccinated PCV2-infected animals and sampled at 15, 16, 17, 18, 19, 20, 22 and 26 weeks of age (post-exposure (PE) period).

The course of PCV2 infection (viremia in serum) was monitored by quantitative PCR (qPCR) and the cellular immune responsiveness was evaluated by flow cytometry (in vivo and after in vitro PCV2 re-stimulation) and an IFN-γ ELISPOT assay. The cellular response detected by ELISPOT was assessed as 1) frequencies of virus-specific IFN-γ secreting cells (SC), 2) IFN-γ responsiveness categories, 3) responsive or non-responsive animals upon definition of a cut-off value, and 4) IFN-γ productivity per cell in single samples and over time.

Serological analyses showed that PRRSV infection had a seroprevalence of 100% at 12 weeks of age, concomitantly with *M. hyopneumoniae* seroconversion, which increased subsequently. Both PRRSV and *M. hyopneumoniae* infections tested by PCR occurred before 15-16 weeks of age and were no longer present at least 4 weeks before PCV2 viremia. Regarding the other above mentioned pathogens, all pigs tested negative. The clinical signs compatible with PCVD were mainly observed between 16 and 23 weeks PV (19-26 weeks of age). Vaccinated animals showed significantly reduced morbidity and mortality (0.2% vs. 9.0%) as well as a significantly higher average daily weight gain (ADWG: +70 g/day) as compared to controls [29].

The study was performed according to the principles of “Good Clinical Practice” and specifically, treatments, housing, husbandry, and feeding conformed to the European Union (EU) Guidelines and identical for both experimental groups. The protocol was approved by the Ethical Committee for Animal Experiments of Parma University.

### PCV2 vaccine, recall virus strain and field isolate

PCV2 vaccination was performed by inoculating one dose of a commercial PCV2a-based subunit vaccine containing the PCV2 capsid (Cap) protein expressed in a baculovirus system (Porcilis® PCV - MSD Animal Health, Whitehouse Station, NJ, USA) suspended in a mineral oil *dl*-α-tocopherol-based adjuvant (MDF, Microsol Diluvac Forte® - MSD Animal Health) administered intramuscularly (2 mL) in the right neck muscle according to the manufacturer’s recommendations.

The in vitro cell re-stimulation for flow cytometry and ELISPOT was performed using a PCV2b virulent strain (I12-11) isolated in 2008 from PMWS-affected pigs in The Netherlands and propagated in PK15 cells.

The field virus infecting the animals under study belonged to the PCV2b genotype as revealed by PCR performed according to Horlen et al. [35].

### Detection of PCV2 viremia in serum

Quantitative PCR (qPCR) was performed on serum to establish the PCV2 status at all sampling times. PCV2 genomic DNA was extracted from 200 μL of serum using TRIzol LS (Invitrogen, San Diego, CA, USA) and suspended in 50 μL of diethylpyrocarbonate (DEPC) water. PCR was performed using primers and probes according to Olvera et al. [36] and a Light-Cycler 1.5 (Roche - Basel, CH). PCV2 titers were expressed as PCV2 genome copies (log_10_)/mL.

### Immunophenotyping and quantification of lymphocyte subsets in the whole blood by flow cytometry

The immunophenotyping and quantification of lymphocyte subsets in the whole blood were performed by flow cytometry as previously described [37-39]. Specifically, cells were double-stained for surface CD3/CD8α, CD4/CD8α, CD3/CD16, CD8β/CD25 and single stained for TCRγ/δ. The following primary antibodies were used: mouse anti-pig-CD3ϵ-PE: clone PPT3, IgG_1K_; mouse anti-pig-CD4α-PE: clone 74-12-4, IgG2_bK_; mouse anti-pig-CD8α-FITC: clone 76-2-11, IgG2_aK_ (Southern Biotech Inc., Birmingham, AL, USA); mouse anti-pig-TCR1-N4 (δ-chain): cell line PGBL22A, IgG_1_ (VMRD Inc., Pullman, WA, USA); mouse anti-pig-CD25 (IL-2 receptor α-chain): clone K231.3B2, IgG_1_ (Serotec, Raleigh, NC, USA); mouse anti-pig-CD8β: cell line PG164A, IgG_2a_ (VMRD); mouse anti-pig-CD16-FITC: clone G7, IgG_1_ (Serotec). TCRγ/δ^+^ and CD8β^+^ cell detection were achieved by a goat F(ab’)_2_ anti-mouse FITC-conjugated antibody (R0480; Dako Cytomation, Glostrup, Denmark). For CD25^+^ cell detection, a goat anti-mouse IgG_1_ PE-conjugate antibody (M32001; Caltag Labs, Burlingame, CA, USA) was used. The analysis was performed using an Epics® XL-MCL cytometer (Beckman-Coulter, Indianapolis, IN, USA) based on PBMC gating after acquisition of at least 10 000 cell events and cell subsets were evaluated based on previous reports. The absolute cell levels (cells/μL) were determined based on the absolute leukocyte counts and lymphocyte percentages [37-40].

### Isolation of porcine PBMC

Porcine peripheral blood mononuclear cells (PBMC) were isolated from blood collected in lithium-heparin by Histopaque-1077® (Sigma, St. Louis, MO, USA) as previously described [41]. PBMC were washed and suspended in complete RPMI-1640 (cRPMI) + 10% FBS (Sigma) and viability was confirmed > 98% by Trypan blue (Sigma). Cell samples not processed immediately were stored in liquid nitrogen in cRPMI + 10% DMSO + 40% FBS. Upon thawing, PBMC were evaluated for viability before being used in the immunological assays.

### Characterization of the PCV2-specific lymphocyte response in PBMC by flow cytometry upon in vitro whole virus re-stimulation

The reactivity of lymphocyte subsets to PCV2 was characterized and quantified by flow cytometry after in vitro PCV2-specific re-stimulation. Briefly, 5 × 10^5^ PBMC were seeded in snap-cap tubes (Sarstedt, Nümbrecht, Germany) and incubated for 48 h in cRPMI + 10% FBS with the PCV2b isolate I12-11 alone (0.25 MOI) or with PCV2 + PHA (5 μg/mL) at 37 °C, 5% CO_2_. PBMC were double-stained with anti-CD4α-PE (clone 74-12-4) and anti-CD8α-FITC (clone 76-2-11) as performed in the whole blood. The analysis was performed after acquisition of at least 10 000 cell events; both resting (lower scatters) and activated PBMC (i.e. lymphoblasts, higher scatters) were considered upon all in vitro conditions. The effective cellular responses to PCV2 or PCV2 + PHA re-stimulation were calculated after subtracting the respective responses of cells incubated in cRPMI-1640 + 10% FBS or with PHA alone in cRPMI-1640 + 10% FBS respectively, and expressed as percentage values [42].

### Combined approach for evaluation of the PCV2-specific IFN-γ secreting cell (SC) response in PBMC

Immune cell activation was also evaluated by quantification of the PCV2-specific IFN-γ secreting cell (SC) frequencies in PBMC, according to Martelli et al. [29] with some modifications regarding the viral re-stimulation. Specifically, PBMC (2 × 10^5^ cells/well) were plated in cRPMI-1640 + 10% FBS and stimulated with the PCV2b isolate I12-11 (range: 0.05 – 0.25 MOI) for 20 h at 37 °C, 5% CO_2_. The IFN-γ secreting cell number was determined by a high-resolution CCD camera-equipped ELISPOT reader and software v.6.0 (Autoimmun Diagnostika, AID®, Straßberg, Germany).

Cell incubation with PHA (10 μg/mL) and in cRPMI-1640 + 10% FBS alone (2 × 10^5^ PBMC/well) were performed as positive and negative controls, respectively. The spot counts in the negative controls were subtracted from the respective counts of the stimulated cells and the immune response was expressed as the number of IFN-γ secreting cells per million of PBMC (IFN-γ SC/10^6^ PBMC).

In addition, the IFN-γ SC response was analyzed by identification of IFN-γ responsiveness categories based on the values of IFN-γ SC/10^6^ PBMC recorded. The categories were defined as follows: 1) no-poor responders: 0-40 SC; 2) low responders: 41-100 SC; 3) intermediate responders: 101-200 SC; 4) high responders: 201-400 SC; 5) very high responders: > 400 SC.

The ELISPOT results were also evaluated upon definition of a cut-off value of 40 IFN-γ SC/10^6^ PBMC in order to classify animals as responders or non-responders; such a value was established based on the observed responses and data in the literature [23].

Based on the CCD camera images and data in the literature [41,43,44], the IFN-γ SC response was evaluated in terms of IFN-γ productivity per cell, taking into account the spot size and intensity distributions in PCV2-stimulated wells.

### Statistical analysis

The statistical analysis for the virological, flow cytometry and IFN-γ ELISPOT continuous data was performed by ANOVA and Dunnett’s test in order to highlight differences between groups and over time throughout the experiment. The analysis of binary (positive/negative) IFN-γ data was performed by the Fisher’s test after determination of a negative-to-positive cut-off value of 40 SC/10^6^ PBMC. All statistical analyses were performed using the SPSS program v.17.0.1 and significance was assessed for *p* < 0.05.

## Results

### PCV2 viremia

**Figure 1:**
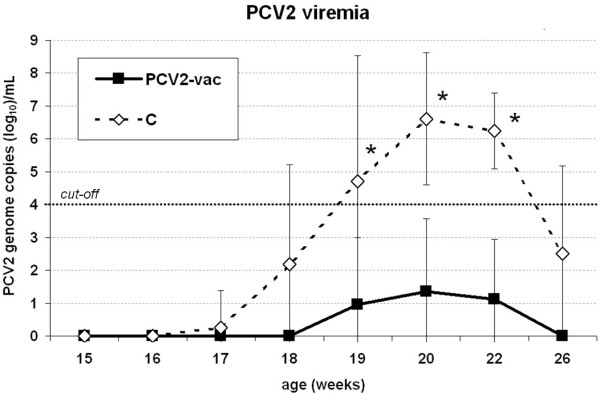
**PCV2 viremia upon PCV2 natural infection.** PCV2 genome copies (log_10_)/mL of serum in PCV2-vaccinated (PCV2-vac) and unvaccinated (C) pigs in the post-exposure (PE) period to PCV2 natural infection (15-26 weeks of age) quantified by qPCR. PCV2-vac pigs were vaccinated at 3 weeks of age using a PCV2a Cap protein-based subunit vaccine. (*): statistically significant difference between groups (*p* < 0.05). The cut-off value of 4 log_10_ (10^4^) PCV2 genome copies/mL is assumed as the lowest limit for a productive infection.

At vaccination (3 weeks of age) and at each time point post-vaccination, all pigs enrolled in the study tested negative for PCV2. Virus could be detected in control pigs only starting from 17-18 weeks up to 26 weeks of age. PCV2 titers gradually increased peaking at 20-22 weeks and then decreased from 22 to 26 weeks (*p* < 0.05). The highest individual variation in the control group was detected at the early phase of infection (18-19 weeks) and upon virus clearance from circulation (26 weeks). The course of PCV2 titers in the control group testifies that the majority of pigs was infected and became viremic or highly viremic. Conversely, PCV2-vac pigs were negative or had very low levels of viremia. The PCV2 titers in controls resulted in being significantly higher than those in the PCV2-vac group from 19 to 22 weeks of age (*p* < 0.05) (Figure 1).

### Lymphocyte subsets in the whole blood

During the post-vaccination (PV) period, no significant difference between PCV2-vaccinated and control animals was detected in the lymphocyte subsets investigated. However, relevant changes were detected over time and characterized by increases of naïve T helper lymphocytes (CD4^+^CD8α^–^ cells: 0.7 × 10^3^ – 1.5 × 10^3^ cells/μL), total and activated cytotoxic T lymphocytes (CD4^–^CD8α^+high^ cells: 1.6 × 10^3^ – 2.6 × 10^3^ cells/μL; CD8β^+^CD25^+^ cells: 25 – 80 cells/μL), circulating memory T helper lymphocytes (CD4^+^CD8α^+low^ cells: 3.5 × 10^2^ – 9.0 × 10^2^ cells/μL) and γ/δ T lymphocytes (TCRγ/δ^+^ cells: 1.4 × 10^3^ – 2.5 × 10^3^ cells/μL). Total and activated NK cells (respectively, CD3^–^CD8α^+^ and CD3^–^CD16^+^ cells) showed more stable values (1.5-2.0 × 10^3^ cells/μL; data not shown).

During the post-exposure (PE) period, slightly higher levels of naïve T helper CD4^+^CD8α^–^ cells were detected in PCV2-vac pigs at weeks 19 and 20 compared to controls (1.3 × 10^3^ cells/μL vs. 1.0 × 10^3^ cells/μL). The course of total cytotoxic T lymphocytes (CD4^–^CD8α^+high^ cells: 2.5 × 10^3^ cells/μL) as well as of total and activated NK cells (CD3^–^CD8α^+^ cells: 1.4 × 10^3^ cells/μL; CD3^–^CD16^+^ cells: 1.2 × 10^3^ cells/μL) were not influenced by PCV2 natural infection in vaccinated and unvaccinated animals. Double positive CD4^+^CD8α^+low^ cells showed highly comparable profiles (0.7-1.1 × 10^3^ cells/μL) in both groups except for higher levels in PCV2-vac pigs compared to controls on week 22 (1.0 × 10^3^ cells/μL vs. 0.7 × 10^3^ cells/μL). TCRγ/δ^+^ cells showed slightly higher levels in vaccinated pigs compared to controls throughout the PE period (3.0-3.7 × 10^3^ cells/μL vs. 2.6-3.0 × 10^3^ cells/μL), while CD8β^+^CD25^+^ (activated cytotoxic T lymphocytes) absolute cell numbers showed increasing and comparable levels in the PCV2-vac and control groups (60 – 130 cells/μL) (data not shown).

### Characterization of the PCV2-specific lymphocyte response in PBMC by flow cytometry upon in vitro whole virus re-stimulation

**Figure 2:**
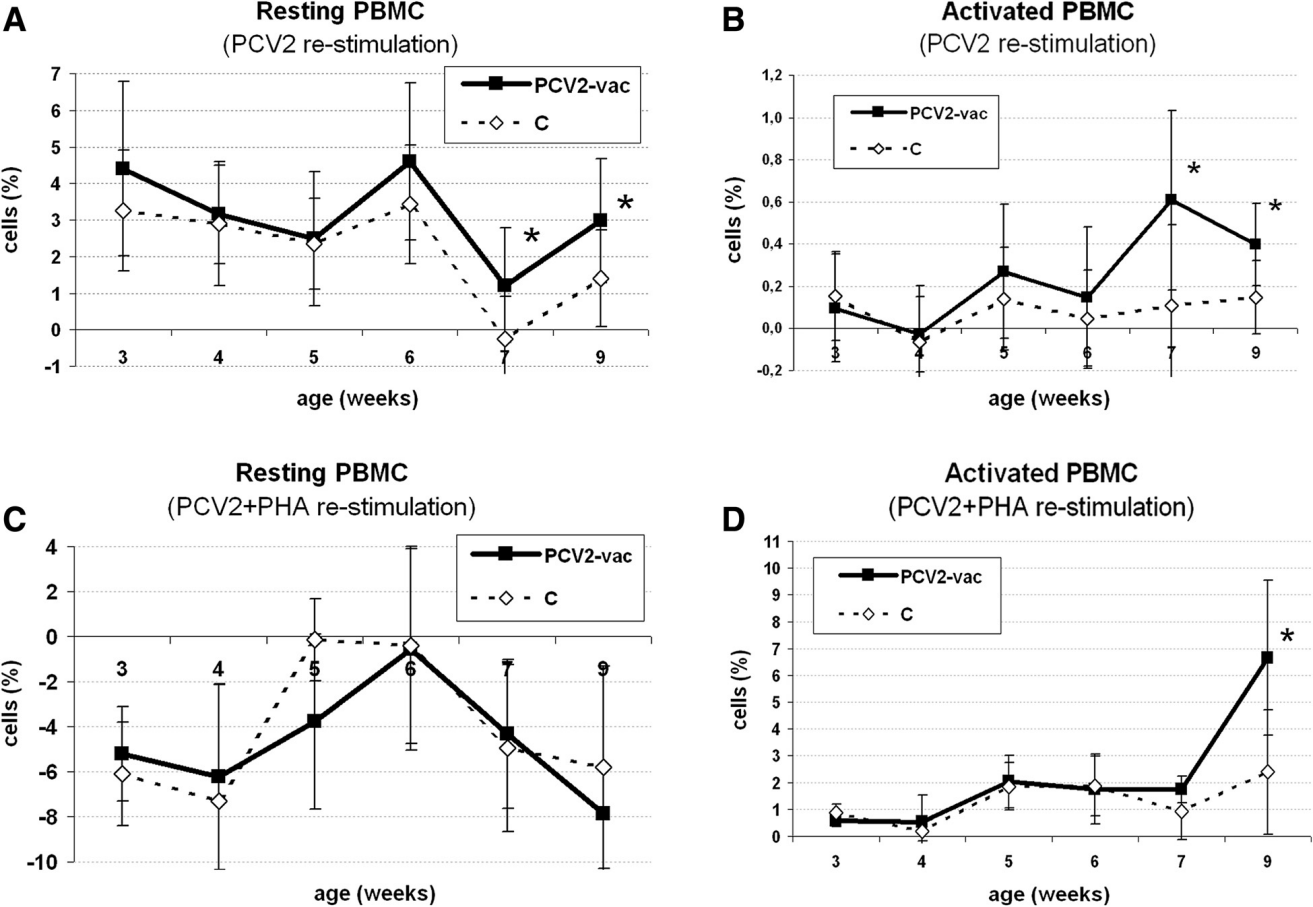
**Total resting PBMC and lymphoblasts upon in vitro stimulation after Cap protein PCV2 vaccination.** Cell levels of resting and activated PBMC in PCV2-vaccinated (PCV2-vac) and control (C) pigs upon 48-h PCV2b **(A, B)** and PCV2b + PHA **(C, D)** in vitro stimulation during the post-vaccination (PV) period. Values are normalized to the corresponding medium (PCV2 stimulation) and PHA-alone (PCV2 + PHA stimulation) control samples, respectively. PCV2-vac pigs were vaccinated at 3 weeks of age using a PCV2a Cap protein-based subunit vaccine. (*): statistically significant difference between groups (*p* < 0.05).

During the PV period, upon PCV2 stimulation, higher levels of both total resting PBMC (Figure 2A) and lymphoblasts (activated PBMC) (Figure 2B) were detected in PCV2-vac pigs at 4 and 6 weeks PV (*p* < 0.05). Upon PCV2 + PHA stimulation, no reactivity and differences between groups were observed in resting PBMC (Figure 2C), whereas the fraction of activated cells characterized by higher forward scatters was highly detectable. Such subpopulation of lymphoblasts showed an increase at 6 weeks PV in PCV2-vac pigs (Figure 2D).

Overall, in vitro stimulation with PCV2 alone led to more restricted activation and proliferation (lower percentages) (Figure 2B), whereas stimulation with PCV2 + PHA induced a strong cell activation to lymphoblasts (higher percentages) (Figure 2D).

Regarding specific T cell subsets, upon PCV2 stimulation, resting total CD4^+^ and CD8α^+^ cells showed negligible stimulation and no differences between groups; CD4^+^CD8α^–^ cells showed very low responsiveness which, however, was higher in vaccinated pigs from 3 weeks PV compared to controls (+0.5-1.5%; *p* < 0.05 at 3 weeks). Resting cytotoxic CD4^–^CD8α^+^ and memory T helper CD4^+^CD8α^+low^ cells did not show significant increases and were not statistically different between groups (data not shown).

**Figure 3:**
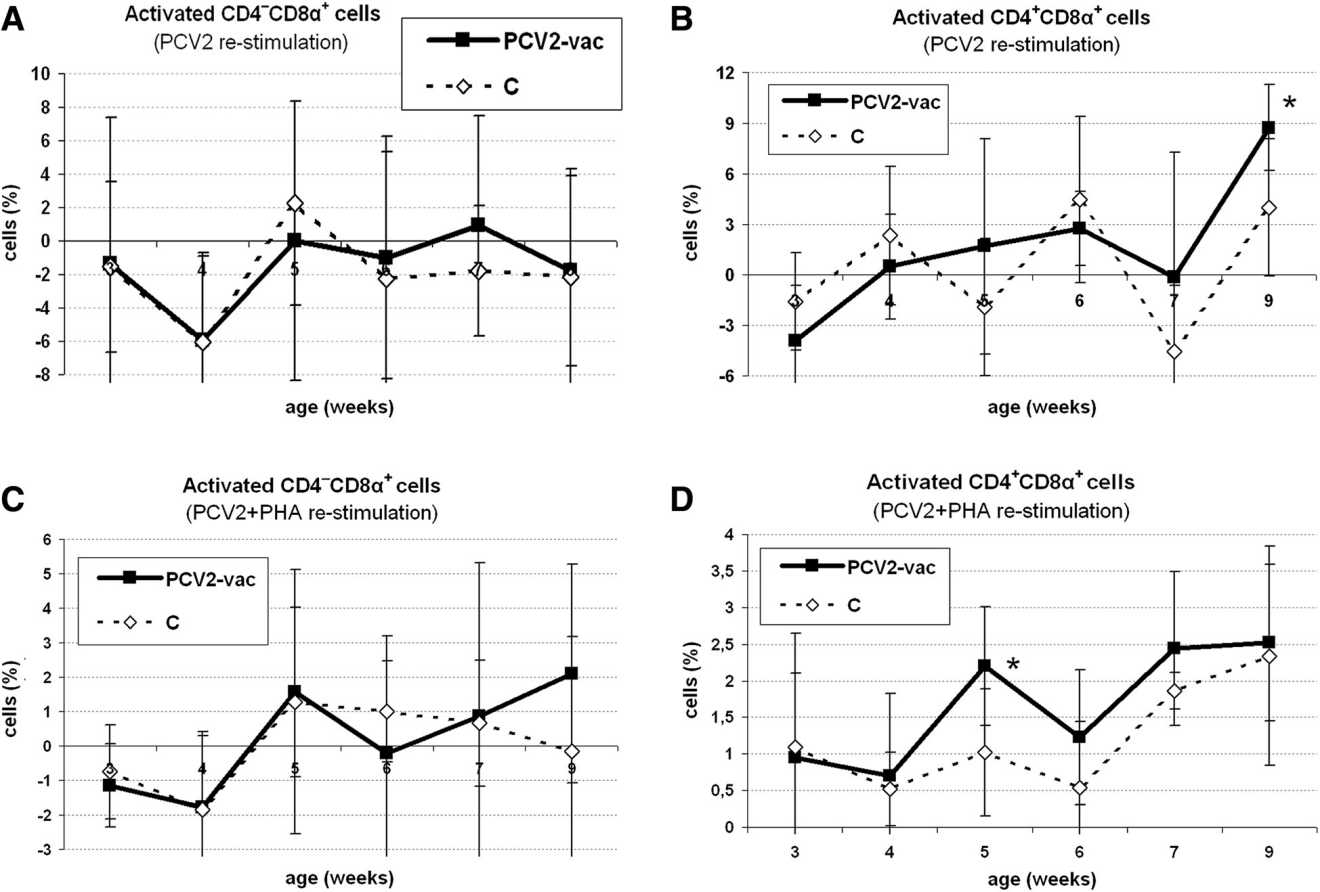
**Cellular immune reactivity of activated T lymphocyte subsets upon in vitro stimulation after Cap protein PCV2 vaccination.** Levels of CD4^–^CD8α^+^ cytotoxic and CD4^+^CD8α^+low^ memory T helper cells in the lymphoblast fraction of PCV2-vaccinated (PCV2-vac) and control (C) pigs upon 48-h PCV2b **(A, B)** and PCV2b + PHA **(C, D)** in vitro stimulation during the post-vaccination (PV) period. PCV2-vac pigs were vaccinated at 3 weeks of age using a PCV2a Cap protein-based subunit vaccine. Values are normalized to the corresponding medium (PCV2 stimulation) or PHA-alone (PCV2 + PHA stimulation) control samples, respectively. (*): statistically significant difference between groups (*p* < 0.05).

In the lymphoblast fraction, activated CD4^–^CD8α^+^ cells did not show relevant differences (Figure 3A) whereas higher reactivity was related to memory CD4^+^CD8α^+low^ T cells at 6 weeks PV (9 weeks of age) (Figure 3B).

Upon PCV2 + PHA stimulation, fluctuating values were detected in both resting total CD4^+^ and CD8α^+^ cells (0-4%) as well as in the specific subsets (0-2.5%); no relevant differences were found between treatment groups (data not shown).

In the PV period, activated total CD4^+^ and CD4^+^CD8α^–^ cells showed comparable responsiveness in both groups whereas CD8α^+^ cells were higher (+3%) in PCV2-vac pigs at 9 weeks of age (6 weeks PV). High individual variation characterized activated CD4^–^CD8α^+^ cells in both groups so that the profiles were comparable, despite a higher mean value in the PCV2-vac group at 9 weeks of age (Figure 3C). Memory CD4^+^CD8α^+low^ cells were slightly higher in the PCV2-vac group from 2 to 4 weeks PV (5-7 weeks of age; *p* < 0.05 at 2 weeks PV) (Figure 3D).

During the post-exposure period, upon PCV2 stimulation, resting PBMC were higher in vaccinated pigs from week 15 to week 18 compared to controls (+0.5-4%); however, the resting lymphocyte specific subsets were not significantly influenced (data not shown).

**Figure 4:**
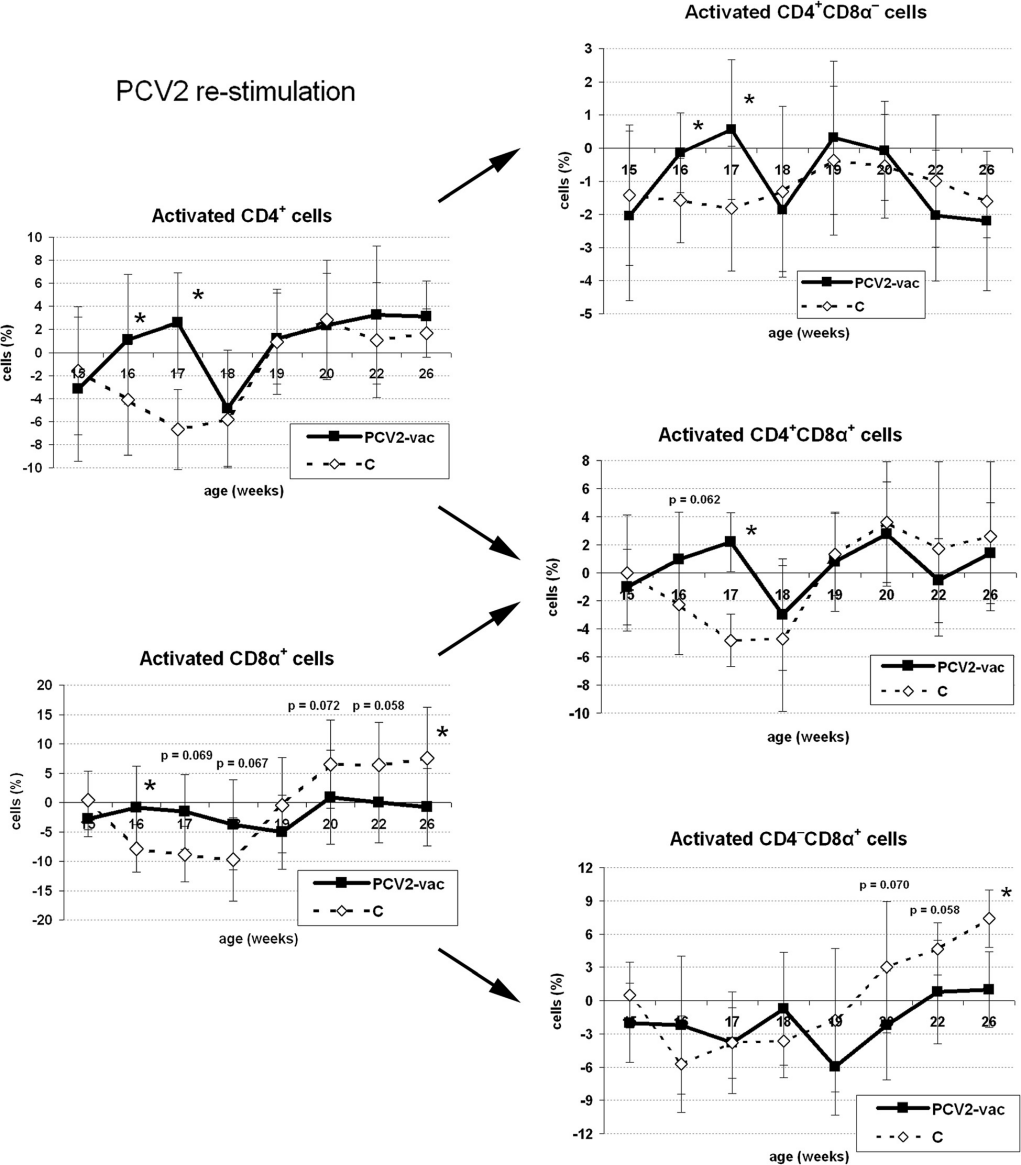
**Cellular immune reactivity of activated T lymphocyte subsets upon in vitro stimulation after PCV2 natural exposure.** Levels of CD4^+^ and CD8α^+^ cell subsets in PCV2-vaccinated (PCV2-vac) and control (C) pigs during the post-exposure (PE) period upon 48-h PCV2b in vitro stimulation. PCV2-vac pigs were vaccinated at 3 weeks of age using a PCV2a Cap protein-based subunit vaccine. Values are normalized to the corresponding medium control samples. (*): statistically significant difference between groups (*p* < 0.05).

Interestingly, despite both groups showing comparable courses of lymphoblast counts, total CD4^+^ and CD8α^+^ cell reactivity was higher in PCV2-vac pigs during the first weeks PE before PCV2 infection occurred (16-17 weeks of age). Specifically, CD4^+^CD8α^–^ naïve T helper cells and especially memory T helper CD4^+^CD8α^+low^ cells were responsible for such different cell reactivity. The increase of total CD8α^+^ cell reactivity from 18-19 weeks of age onwards was concomitant with the response of the cytotoxic CD4^–^CD8α^+^ subset, which resulted in being more intense in unvaccinated infected pigs (Figure 4).

During the PE period, upon PCV2 + PHA stimulation, resting PBMC did not show any significant changes over time and between groups; resting total CD4^+^ and CD8α^+^ cells showed a responsiveness of 0-1% and 2-7%, respectively. Activated PBMC increased over time (0.5% – 3%) in both groups and showed comparable levels; the changes were due to changes of CD8α^+^ cells (0-5%), specifically CD4^–^CD8α^+^ cells during the first 2-3 weeks (0-4%). Reactive CD4^+^ cells were composed of activated CD4^+^CD8α^–^ cells both in PCV2-vac and control pigs (data not shown).

### PCV2-specific IFN-γ secreting cell (SC) response in PBMC

During the post-vaccination (PV) period, the response quantified as frequencies of IFN-γ SC highlighted that immune responsiveness was elicited in PCV2-vac pigs early after vaccine inoculation; a statistically different response between PCV2-vac and control animals was observed from 2 weeks PV (5 weeks of age) onwards, up to 6 weeks PV (9 weeks of age) (*p* < 0.05). However, some pigs categorized as low (41-100 SC) and intermediate (101-200 SC) responders showed a detectable response already at 1 week PV. The response was negligible in controls throughout the PV period (data not shown).

Specifically, since PCV2-vac pigs showed high individual variation, several IFN-γ responsiveness categories were identified. Before vaccination, all vaccinated animals showed frequencies of 0-40 IFN-γ SC/10^6^ PBMC identifying no-poor responders, whereas pigs showing a response of 41-200 IFN-γ SC/10^6^ PBMC were detected already at 1 week PV. The maximum response was observed between 2 and 6 weeks PV and was characterized by a major fraction of high (201-400 IFN-γ SC/10^6^ PBMC) and very high (> 400 IFN-γ SC/10^6^ PBMC) responder animals. The control pigs were distributed in the no-poor response category throughout the PV period.

The analysis of the ELISPOT response based on the definition of a cut-off value of 40 IFN-γ SC/10^6^ PBMC allowed determining a statistically significant response from 1 week PV (2/10 vs. 0/10 positive pigs; *p* < 0.05) onwards in PCV2-vac pigs compared to controls. In particular, the majority of vaccinated pigs showed a significant response between 2 (8/10) and 3 (9/10) weeks PV (*p* < 0.05). The SC response upon cut-off determination showed the same trend as upon analysis of mean values (data not shown).

**Figure 5:**
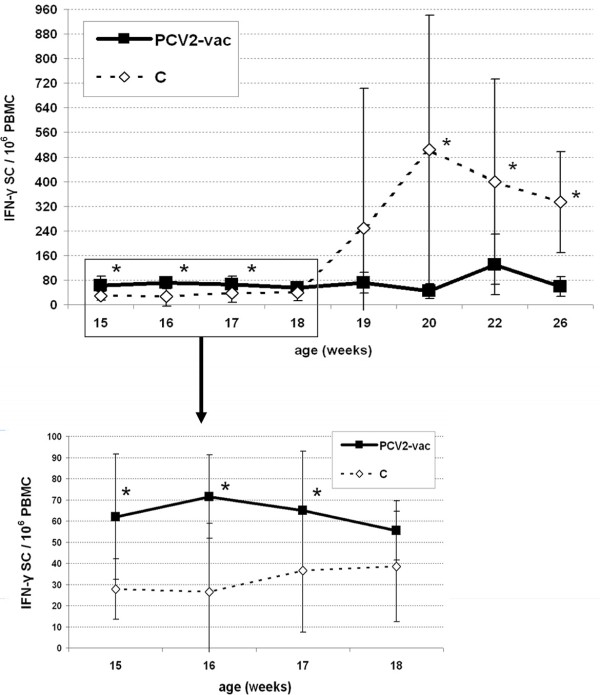
**Frequencies of PCV2-specific IFN-γ secreting cells (SC) upon PCV2 natural exposure.** Levels of IFN-γ SC in PBMC of PCV2-vaccinated (PCV2-vac) and unvaccinated (C) pigs in the post-exposure (PE) period. The magnified graph highlights the profiles of the two groups before the onset of significant PCV2 viremia in the PE period. PCV2-vac pigs were vaccinated at 3 weeks of age using a PCV2a Cap protein-based subunit vaccine. PBMC were ex vivo re-stimulated for 20 h with 0.25 MOI of a whole PCV2b strain. (*): statistically significant difference between groups (*p* < 0.05).

During the post-exposure (PE) period, the IFN-γ SC response in PCV2-vac pigs was higher than in controls up to week 3 (18 weeks of age), showing mean values of 60-70 IFN-γ SC/10^6^ PBMC. During these weeks, the control group showed mean frequencies of 30-40 IFN-γ SC/10^6^ PBMC. Afterwards, the levels in controls significantly increased and reached higher levels from week 5 to week 11 PE (20-26 weeks of age; *p* < 0.05). Vaccinated pigs showed a significant temporary increase on week 7 PE (22 weeks of age; *p* < 0.05) (Figure 5).

**Figure 6:**
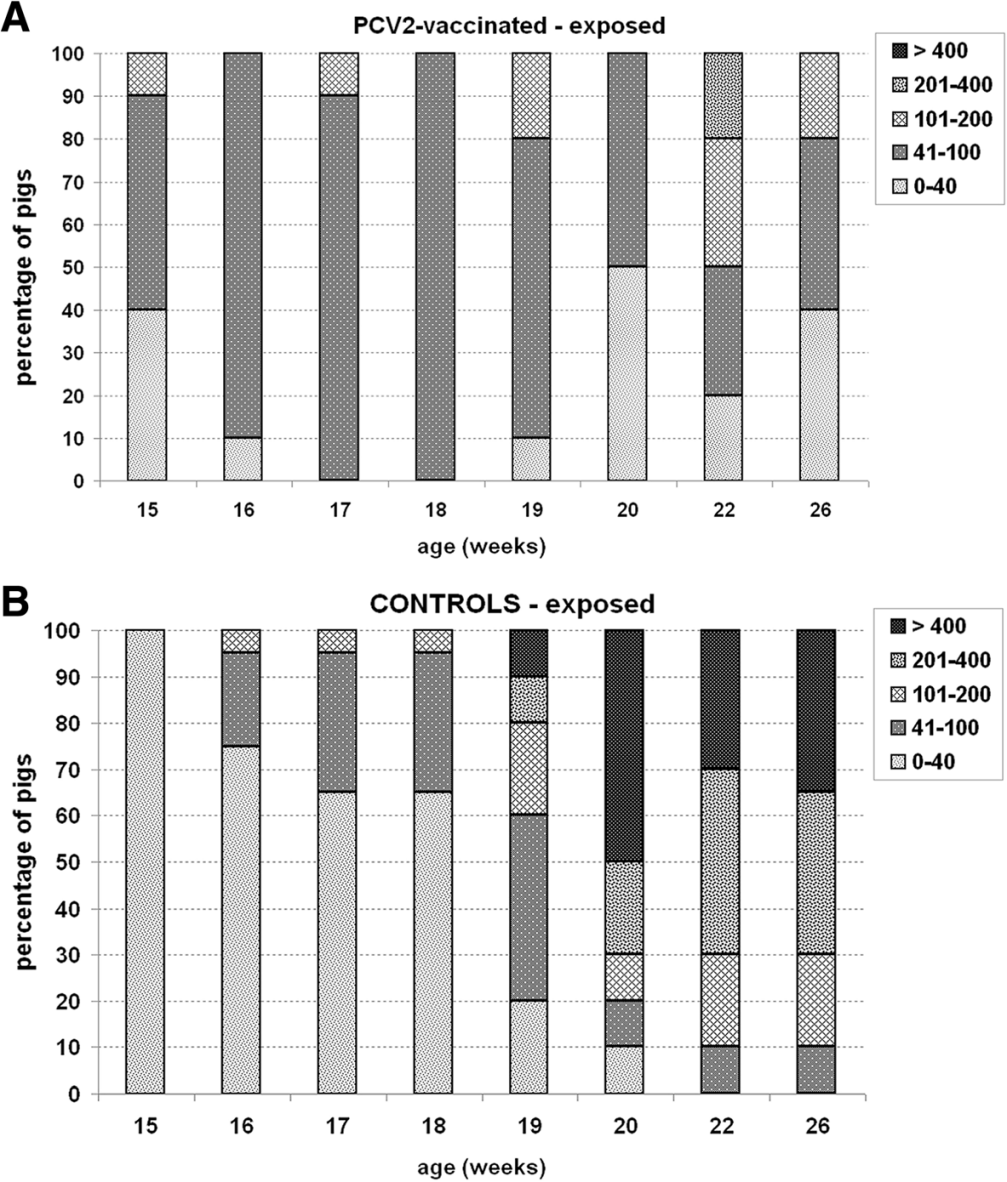
**PCV2-specific IFN-γ responsiveness categories upon PCV2 natural exposure.** Category distribution in PBMC of **(A)** PCV2-vaccinated (PCV2-vac) and **(B)** unvaccinated control (C) pigs in the post-exposure (PE) period to PCV2 natural infection (15-26 weeks of age). PCV2-vac pigs were vaccinated at 3 weeks of age using a PCV2a Cap protein-based subunit vaccine. PBMC were ex vivo re-stimulated for 20 h with 0.25 MOI of a whole PCV2b strain.

The analysis performed by IFN-γ responsiveness categories highlighted that the most represented fraction in vaccinated animals was characterized by relatively low responders (41-100 IFN-γ SC/10^6^ PBMC) at almost all time points. A minor fraction of intermediate (101-200 IFN-γ SC/10^6^ PBMC) and high (201-400 IFN-γ SC/10^6^ PBMC) responders was also detected both early (10%) and late (20-30%) after exposure, respectively (Figure 6A).

Conversely, in control pigs, the category distribution drastically changed after the onset of infection in favor of large fractions of high and very high responders after 18 weeks of age. At 22-26 weeks of age, no/poor responders were not detected in controls and fractions ranging from 10% to 50% of very high responders were found (Figure 6B).

**Figure 7:**
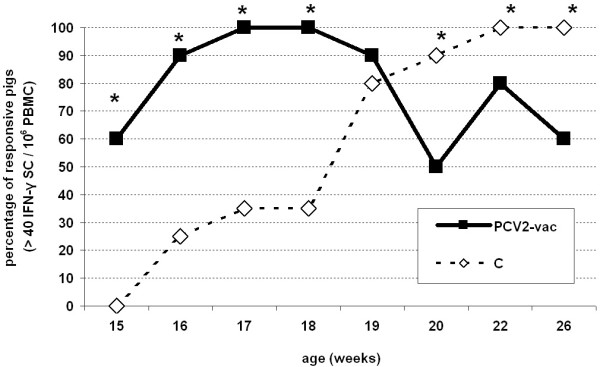
**PCV2-specific ELISPOT positivity upon PCV2 natural exposure.** Percentages of responsive animals evaluated upon definition of a cut-off value of 40 IFN-γ SC/10^6^ PBMC in PCV2-vaccinated (PCV2-vac) and unvaccinated (C) pigs in the post-exposure (PE) period to PCV2 natural infection (15-26 weeks of age). PCV2-vac pigs were vaccinated at 3 weeks of age using a PCV2a Cap protein-based subunit vaccine. PBMC were ex vivo re-stimulated for 20 h with 0.25 MOI of a whole PCV2b strain. (*): statistically significant difference between groups (*p* < 0.05).

ELISPOT data analyzed using the cut-off value showed that the PCV2-vac group had a significantly higher fraction of responding pigs before the onset of PCV2 viremia (15-18 weeks of age) ranging between 60% and 100% compared to controls (*p* < 0.05), whereas unvaccinated pigs had no relevant response at 15 weeks of age and only a minor fraction of responding animals between 16 and 18 weeks of age (25% and 35%). Upon infection, controls showed a relevant increase of responsiveness up to 100% at 22-26 weeks of age while PCV2-vac pigs ranged between 50% and 80% (*p* < 0.05) (Figure 7).

The IFN-γ SC response was additionally evaluated as IFN-γ productivity per cell depending on the amount of IFN-γ secreted by a single cell.

During the PV period, the early responding pigs (response at 1 week PV) were characterized by low frequencies of IFN-γ SC generally associated with low secretion per cell, which means small and/or low intensity spots independently from the PCV2 amount used as recall antigen.

**Figure 8:**
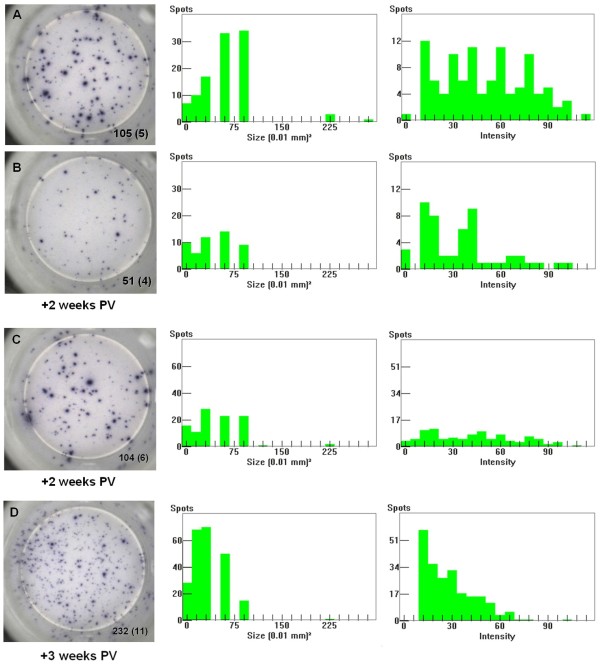
**Differential IFN-γ productivity after Cap protein PCV2 vaccination.** Comparison of two representative samples of PCV2-vaccinated pigs characterized by higher **(A)** and lower **(B)** PCV2-specific IFN-γ productivity per cell, that is larger and smaller spot sizes depending on the amount of IFN-γ secreted by ex vivo PCV2-stimulated cells. The responses shown were observed at 2 weeks after PCV2 vaccination (PV) upon re-stimulation with a PCV2b strain (0.25 MOI). A representative transient increase of IFN-γ productivity per cell is shown in a PCV2-vaccinated pig at 2 weeks **(C)** and 3 weeks **(D)** PV. The numbers are relative to the spots counted in the stimulated well shown (2 × 10^5^ PBMC) and (in brackets) the mean of the corresponding unstimulated negative control wells (2 × 10^5^ PBMC). The spot size and spot intensity distributions are shown as histogram plots on a linear scale. PCV2-vac pigs were vaccinated at 3 weeks of age using a PCV2a Cap protein-based subunit vaccine.

The in-depth visual monitoring of the post-vaccination responses allowed detecting differential behaviors in PCV2-vac pigs. At 2 weeks PV, some animals showed higher frequencies of IFN-γ SC characterized by much higher IFN-γ productivity per cell (Figures 8A and B). In addition, some samples showed a strong heterogeneity of IFN-γ productivity at a specific time point and a shift from higher to lower productivity associated with increased frequencies between 2 and 3 weeks PV (Figures 8C and D).

**Figure 9:**
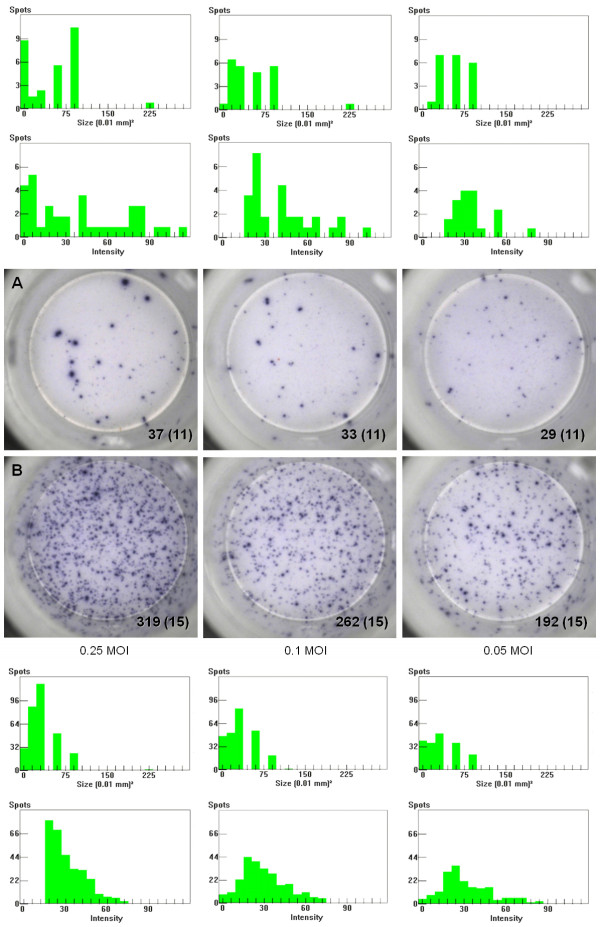
**Differential PCV2-specific IFN-γ secretion responses upon PCV2 natural exposure.** Representative responses of one PCV2-vaccinated **(A)** and one unvaccinated **(B)** pig at 19 weeks of age (+4 weeks post-exposure (PE) to PCV2 natural infection) characterized respectively by stable IFN-γ SC number and increased/higher IFN-γ productivity per cell **(A)** or increased cell number and stable/lower IFN-γ productivity per cell **(B)** depending on the amount of PCV2b used as ex vivo recall antigen. The numbers are relative to the spots counted in the stimulated well shown (2 × 10^5^ PBMC) and (in brackets) the mean of the corresponding unstimulated negative control wells (2 × 10^5^ PBMC). The spot size and spot intensity distributions are shown on a linear scale. PCV2-vac pigs were vaccinated at 3 weeks of age using a PCV2a Cap protein-based subunit vaccine.

The evaluation of the IFN-γ productivity during the PE period, and specifically after PCV2 natural infection, highlighted different features when comparing PCV2-vac and control pigs: 1) in some PCV2-vac animals, a lower IFN-γ SC response was characterized by a stable cell frequency and increase of IFN-γ productivity depending on the recall PCV2 antigen amount (Figure 9A); 2) in some control animals, a much higher SC response was characterized by an increasing cell frequency and stably lower IFN-γ productivity depending on the recall PCV2 antigen amount (Figure 9B).

## Discussion

The study was performed in maternally-immune piglets intramuscularly vaccinated for PCV2 by administration of a Cap-based subunit vaccine and in unvaccinated animals, subsequently exposed to PCV2 natural infection. The high degree of protection against severe PCVD clinical signs observed after infection in vaccinated pigs was demonstrated by the significant reduction of morbidity and mortality associated with PCV2 positivity and PCVD-compatible lesions as well as by the improvement of the ADWG [29].

The changes observed over time during the PV period in both groups related to the CD4^+^ T helper (naïve CD4^+^CD8α^–^ and memory CD4^+^CD8α^+low^) and total/activated CD8^+^ cytotoxic (CD4^–^CD8α^+high^, TCRγ/δ^+^, CD8β^+^CD25^+^) subsets can be attributed to the physiological development of the immune competence in growing piglets [37].

The exposure to PCV2 natural infection was evident both as high viremia levels and PCVD clinical signs from 19 weeks of age in unvaccinated animals. During the PE period, the slightly but not significantly higher levels of CD4^+^CD8α^–^ T helper cells and γ/δ T lymphocytes in the PCV2-vac group were attributed to changes independent of infection. Under the conditions of this study, PCVD was not associated with a significant modulation of these peripheral cell subsets involved in sustaining the Th1-biased anti-viral immunity upon infection. This can be due to the different extent of negative immune modulation induced by PCV2 in infected pigs depending on different field conditions [12,18-20,25].

Conversely, the involvement of both CD4^+^ and CD8^+^ cells in terms of lymphoproliferative response upon PCV2 infection appeared to be important to evaluate, as previously demonstrated under experimental conditions [45]. The cellular responsiveness to PCV2 antigen was evaluated upon in vitro stimulation of PBMC with whole PCV2 alone or in the presence of PHA as a mitogenic stimulating factor, since it is known that mitogens can synergistically up-regulate several cell responses to PCV2 due to their IFN-α/IFN-γ-dependent lymphoproliferative effects [4,5,46,47].

Post-vaccination data showed that the PCV2 in vitro recall had a stimulatory effect on both resting PBMC and PBMC activated to lymphoblasts at 4-6 weeks PV, suggesting a delayed and not intense induction of immune cells able to respond when stimulated by the viral antigen. This responsiveness seemed to be sustained by CD4^+^ cells, specifically T helper naïve CD4^+^CD8α^–^ and memory CD4^+^CD8α^+low^ cells. The recall of memory cells is particularly important since this subset is involved in the regulation of the anti-viral response by IFN-γ secretion and virus clearance when infection occurs [40].

The addition of PHA induced higher levels of responding cells in the activated fraction only, suggesting a detectable influence on proliferating previously primed cells of vaccinated animals. These in vitro conditions highlighted higher CD4^–^CD8α^+^ cells at 6 weeks PV, thus also pointing out a possible involvement of cytotoxic cells.

It is noteworthy that the absence of significant differences or erratic responses during the early time points after vaccination could have been influenced by the ability of the whole virus used as in vitro recall antigen to induce inhibition of immune responsiveness that counteracted cell activation [8,33,48].

During the PE period, the levels of activated CD4^+^ and CD8α^+^ cells in vaccinated pigs were significantly higher than in controls during the first weeks before infection (15-17 weeks); these subsets were mainly constituted of naïve and memory T helper cells able to respond to PCV2. In vaccinated animals, memory cells can be involved in efficiently coping with infection and preventing the disease. It is well known that memory lymphocytes produce IFN-γ, thus driving the Th1 response, activating cytotoxic effector cells to eliminate infected cells, and promoting virus-specific antibody production [40].

In fact, in vaccinated pigs, the onset of PCV2 infection was promptly counteracted so that a significant viremia was detected in one pig only at one time point (10^5^ PCV2 genome copies/mL). High variation was observed in cell counts in both groups, suggesting that the in vitro interaction with the virus is variable since both monocytes and lymphocytes can be differentially infected [3,4,47,49].

The in vitro inhibitory effect of whole PCV2 stimulation seemed to be evident in unvaccinated animals at the time points preceding infection since negative percentage values represent lower values in the stimulated samples compared to unstimulated PBMC used as corresponding internal controls. In control animals, upon establishment of infection (19-26 weeks), higher percentages of CD8α^+^ and CD4^–^CD8α^+^ cells in the absence of significantly higher values of memory CD4^+^CD8^+low^ cells demonstrate that total CD8^+^ cells were mainly constituted by cytotoxic effector cells (likely NK cells, γ/δ T and cytotoxic T lymphocytes) which were not previously primed.

PCV2 vaccination elicited an early and significant IFN-γ SC response; the significant response was confirmed by cut-off analysis in agreement with previous data by Pérez-Martín et al. [23]. The early responding pigs showed low IFN-γ productivity per cell (spot size and intensity depending on the amount of cytokine secreted by single cell) suggesting that this early responsiveness was associated with low secretion T cells.

The response strongly increased during the following weeks, reaching average frequencies of 200 SC/10^6^ PBMC due to very high responder pigs, differently from what was observed under experimental conditions with conventional and SPF piglets [17,23], testifying that vaccination induced a very strong response in conventional pigs. The response could be properly detected using a PCV2b isolate as in vitro recall antigen in PCV2a Cap-based vaccinated pigs; this further supports cross-reactivity in antigen recognition between different genotypes [24,33].

This study first demonstrates that a marked increase of IFN-γ productivity per cell concomitant with the increase of IFN-γ SC frequency is a mechanism observed in pigs as response to vaccination with a PCV2 Cap-based subunit vaccine similarly to what is documented with vaccinia-virus in humans [43]. Since these highly productive cells are often considered to be the most protective, this is an important feature and marker to evaluate by ELISPOT. This behavior was transient as in humans, not being evident in cells from vaccinated animals at 3 weeks of age; also in conventional pigs, this suggests that increased productivity is related to recent in vivo T cell activation rather than being a stable condition.

The increase of IFN-γ productivity detected in some vaccinated pigs after infection strengthens what was observed after vaccination and may represent an important mechanism sustaining the efficiency of the immune response.

Before infection (15-18 weeks), PCV2-vac pigs showed significantly higher SC levels than controls. The minor fraction of responsive pigs in the unvaccinated group may be due to an earlier infection in some animals, although unspecific responses cannot be completely ruled out.

In PCV2-vac pigs, the parallel higher frequencies of IFN-γ secreting cells and percentages of virus-specific CD4^+^CD8^+low^ T cells at 15-18 weeks suggest that vaccination sustained long-lasting immune memory cells that are able to proliferate and potentially responsible for IFN-γ secretion upon re-exposure to the PCV2 antigen.

When infection and clinical signs occurred, unvaccinated pigs showed a significant increase of IFN-γ SC frequencies, with the highest fraction of very high responders (> 400 SC) at 2 weeks post-infection. This study highlights that in the field, on the contrary to what was observed under controlled conditions [17,23], a wide distribution in all the responsiveness categories was found in control pigs testifying the extremely variable response to primary PCV2 infection, especially during the first weeks of infection.

In control pigs, IFN-γ secreting cells increased simultaneously with CD4^–^CD8α^+^ effector cells, as reported under experimental conditions (SPF piglets, asymptomatic infection [13]). Moreover, the spread of infection and onset of clinical signs in control pigs support the hypothesis of an altered immune responsiveness, since the high levels of IFN-γ SC did not associate with efficient viral clearance; this latter condition could be related to a negative immunomodulation by the induction of IL-10, as previously reported in severely diseased animals [10].

Vaccinated pigs showed a lower and more homogeneous response characterized by a large fraction belonging to the low/intermediate responsiveness categories as observed under similar field conditions [30]. As discussed above, an important feature observed during infection was that the lower IFN-γ SC response in some vaccinated animals compared to the high response in the majority of unvaccinated pigs was associated with increased productivity per cell instead of increased cell frequency. This feature is worth being further investigated since no clear association between post-vaccination and/or post-infection IFN-γ productivity and protection was found.

In conclusion, under the conditions of this study, the administration of a PCV2 subunit vaccine induced a long-lasting immunity sustained by reactive CD4^+^CD8^+^ memory T cells and IFN-γ secreting cells, which were associated with the prevention or reduction of infection and clinical signs. The extent and duration of this cellular reactivity can be fundamental to drive an efficient Th1-biased biased response to infection, thus representing an important feature for the evaluation of immune protection induced by vaccination in the field.

## Abbreviations

A. pleuropneumoniae: *Actinobacillus pleuropneumoniae*; ADV: Aujeszky’s disease virus; ADWG: Average daily weight gain; ANOVA: Analysis of variance; Cap: Capsid; CCD: Charge coupled device; CD: Cluster of differentiation; CTL: Cytotoxic T lymphocytes; DC: Dendritic cells; DEPC: Diethylpyrocarbonate; DMSO: Dimethyl sulfoxide; ELISPOT: Enzyme-linked immunospot; FBS: Fetal bovine serum; FITC: Fluorescein isothiocyanate; γ/δ: Gamma/delta; IFN-α: Interferon-alpha; IFN-γ: Interferon-gamma; Ig: Immunoglobulin; IL: Interleukin; IM: Intramuscularly; M. hyopenumoniae: *Mycoplasma hyopneumoniae*; MDF: Microsol diluvac forte^®^; MOI: Multiplicity of infection; NK: Natural killer; PBMC: Peripheral blood mononuclear cells; PCV2: Porcine circovirus type 2; PCV2a: Porcine circovirus type 2 - genotype “a”; PCV2b: Porcine circovirus type 2 - genotype “b”; PCVD: Porcine circovirus associated disease; PE: Post-exposure; PHA: Phytohemagglutinin; PK: Porcine kidney; PMWS: Post-weaning multisystemic wasting syndrome; PRRSV: Porcine reproductive and respiratory syndrome virus; PV: Post-vaccination; qPCR: Quantitative polymerase chain reaction; PE: Phycoerythrin; RPMI: Roswell park memorial institute; SC: Secreting cells; SIV: Swine influenza virus; SPF: Specific pathogen-free; SWC: Swine workshop cluster; TCR: T cell receptor; Th: T helper; TNF-α: Tumor necrosis factor-alpha; VNA: virus neutralizing antibodies.

## Competing interests

The authors declare that they have no competing interests.

## Authors’ contributions

All authors equally contributed to design the study and to evaluate and discuss the results. All authors read and approved the final manuscript.
